# Interplaying role of healthcare activist and homemaker: a mixed-methods exploration of the workload of community health workers (Accredited Social Health Activists) in India

**DOI:** 10.1186/s12960-020-00546-z

**Published:** 2021-01-06

**Authors:** Anand Kawade, Manisha Gore, Pallavi Lele, Uddhavi Chavan, Hilary Pinnock, Pam Smith, Sanjay Juvekar, for the RESPIRE collaboration, Steve Cunningham, Farzana Khan, Colin Simpson, David Weller, Nazimuddin Zulma, Andrew Morris, Roberto Rabinovitch, Tabish Hazar, Li Ping Wong, Pam Smith, Rita Isaac, Parag Khataokar, Osman Yusuf, Shahida Yusuf, Liz Grant, Harry Campbell, Aziz Sheikh

**Affiliations:** 1 Vadu Rural Health Program, King Edward Memorial Hospital Research Centre (KEMHRC), Rasta Peth, Pune, Maharashtra 411011 India; 2 Symbiosis Institute of Health Sciences, Symbiosis International (Deemed) University, Lavale, Mulshi, Pune, Maharashtra 411011 India; 3 NIHR Global Health Research Unit on Respiratory Health (RESPIRE) Usher Institute, University of Edinburgh, Doorway 3, Medical School, Teviot Place, Edinburgh, EH8 9AG United Kingdom; 4 Nursing Studies, School of Health in Social Science, NIHR Global Health Research Unit on Respiratory Health (RESPIRE), University of Edinburgh, Teviot Place, Edinburgh, EH8 9AG United Kingdom

**Keywords:** ASHA, Workload, Community health worker

## Abstract

**Background:**

Globally, community health workers (CHWs) are integral contributors to many health systems. In India, Accredited Social Health Activists (ASHAs) have been deployed since 2005. Engaged in multiple health care activities, they are a key link between the health system and population. ASHAs are expected to participate in new health programmes prompting interest in their current workload from the perspective of the health system, community and their family.

**Methods:**

This mixed-methods design study was conducted in rural and tribal Primary Health Centers (PHCs), in Pune district, Western Maharashtra, India. All ASHAs affiliated with these PHCs were invited to participate in the quantitative study, those agreeing to contribute in-depth interviews (IDI) were enrolled in an additional qualitative study. Key informants’ interviews were conducted with the Auxiliary Nurse Midwife (ANM), Block Facilitators (BFF) and Medical Officers (MO) of the same PHCs. Quantitative data were analysed using descriptive statistics. Qualitative data were analysed thematically.

**Results:**

We recruited 67 ASHAs from the two PHCs. ASHAs worked up to 20 h/week in their village of residence, serving populations of approximately 800–1200, embracing an increasing range of activities, despite a workload that contributed to feelings of being rushed and tiredness. They juggled household work, other paid jobs and their ASHA activities. Practical problems with travel added to time involved, especially in tribal areas where transport is lacking. Their sense of benefiting the community coupled with respect and recognition gained in village brought happiness and job satisfaction. They were willing to take on new tasks. ASHAs perceived themselves as ‘voluntary community health workers’ rather than as ‘health activists”.

**Conclusions:**

ASHAs were struggling to balance their significant ASHA work and domestic tasks. They were proud of their role as CHWs and willing to take on new activities. Strategies to recruit, train, skills enhancement, incentivise, and retain ASHAs, need to be prioritised. Evolving attitudes to the advantages/disadvantages of current voluntary status and role of ASHAs need to be understood and addressed if ASHAs are to be remain a key component in achieving universal health coverage in India.

## Background

Globally, community health workers (CHWs) contribute to achieving universal health care coverage; a key target for meeting Millennium Development Goals [1]. The World Health Organization’s (WHO) definition of a CHW is a person living and working within the local community, being endorsed by the health system, but not necessarily part of it and having shorter training than professional workers [2]. The scope of CHW’s work often encompasses large-scale programmes addressing local health problems in rural and remote areas of low- and middle-income countries (LMICs) [3].

In 2005, the National Rural Health Mission of the Government of India launched an Accredited Social Health Activists (ASHA) programme to facilitate accessible, affordable and quality healthcare to rural populations. ASHA is a female resident of the village, educated at least to VIIIth grade (though may not be enforced in tribal areas) who receives 23 days training over a year and on-going refresher courses [4]. Currently, there are more than one million (1,047,324) ASHAs countrywide, covering the entire population except for the state of Goa. In rural areas there is about one ASHA per 879 population, but with wide intra-interstate variations [5, 6]. A key link between health system and population, and as a multitasker, ASHAs took on the “social Activist “roles of health educator, healthcare services facilitator with evidence of a positive impact on healthcare-seeking behaviour, family planning, antenatal care and care in childbirth [7–13].

Building on this success, policymakers have upscaled ASHAs’ involvement in an increasing range of health-related activities and interventions [14–18] as well as implementing governmental public health schemes and surveys. The Indian National Program for Prevention and Control of Cancer, Diabetes, Cardiovascular Disease and Stroke ((NPCDCS) involves ASHAs in screening, early detection, referral and community mobilisation of Non-Communicable Diseases (NCDs) [19]. Thus, ASHAs are engaged in almost 30 different activities which sometimes means non-health-related tasks might take priority over health-related issues [5, 6, 17].

The role of ASHA is therefore evolving and demands an up-to-date comprehensive assessment of the workload, incentives [20] and understanding of the work profile from the perspectives of the health system, community and the ASHA herself in order to guide successful implementation as well as sustainability of the programme. Previous evaluations, many commissioned by the National ASHA Mentoring Group [8], provide qualitative exploration or quantitative assessment [12–15] of workload, often in one specific context or context of maternal/child health tasks [21–25]. We had a broad interest in both: range of tasks and different situations in which ASHA works and the changing context in which their role is interpreted. We therefore used a mixed-methods approach to assess and explore ASHAs’ perspectives of their workload alongside that of local healthcare colleagues in both rural and tribal contexts.

## Methods

The study was conducted in two Primary Health Centres (PHCs), one rural and the other tribal (often remote, a defined inhabitant in India with shared ancestry and traditions) in Pune district of Western Maharashtra, India during September 2018 to March 2019.

### Study area and context

Over the last five decades, Vadu Rural Health Program’s research and implementation activities have developed a good collaboration with the local healthcare systems which facilitated the selection and recruitment of the PHCs functionaries and ASHAs.

**Fig. 1:**
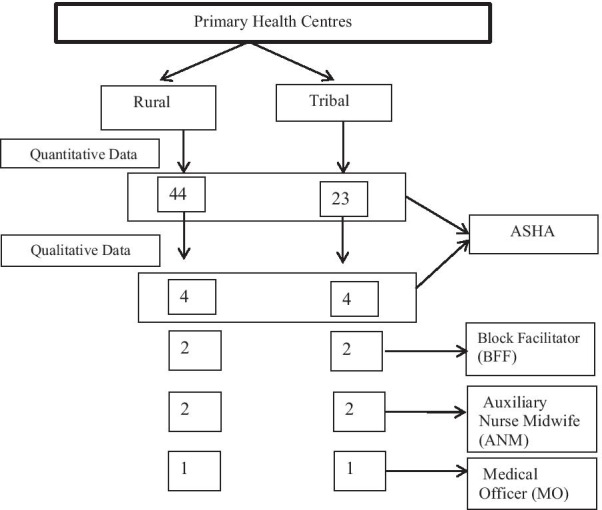
_Flowchart showing distribution of study participants. Block Facilitator (BFF)- has to support ASHA activities. He/she is the person to be in direct touch with the frontline ASHA workers and providing supportive supervision is the main role [36]. Auxiliary Nurse Midwife (ANM)- are regarded as the grass-roots workers in the health organisation pyramid. Their services are considered important to provide safe and effective care to village communities. The role may help communities achieve the targets of national health programmes [37]. Medical Officer (MO)- is in charge of the Primary Health centre he/she takes an active role in overseeing medical care of patients and functions performed by medical staff. He/she may participate directly in care when services are implemented. The. MO may also help assess and diagnose needs and plan of action for individuals and families [38]_

The rural PHC is located in an agricultural area with increasing industrialisation. This high-density population had multiple sources of income, travel and communication facilities. Many private hospitals and clinics provide multiple choices for healthcare. In contrast, the tribal PHC is located in hilly terrain where a sparse, culturally homogenous population had fewer sources of income and poor access to travel and communication and almost complete reliance on public healthcare facilities. Selecting these diverse PHCs enabled us to study whether these contextual differences affected the workload perception of ASHAs. The distribution of study participants is described in Fig. 1.

### Quantitative methods

The quantitative data were collected using paper-based self-administered questionnaires. All 67 ASHAs (44 from Rural PHC; 23 from Tribal PHC) responded when approached during their routine monthly meetings and vaccination camps. The closed questions were on socio-demographic profile, time spent on ASHA work and travel, perceptions of workload and its impact on them and their family, opinions about remuneration, job satisfaction and family support. (Additional file 1: Study questionnaire).

### Qualitative methods

Qualitative data were collected using in-depth interviews. We purposively sampled eight ASHAs, four from each of the rural and tribal PHCs, based on diversity of experience, educational background and other paid work. We included ASHAs having at least 5 years standing so that they had experience of ASHA work in the community. We also interviewed the two Medical Officers (MO), four Auxiliary Nurse Midwives (ANM) and four Block Facilitators (BFF) from the same PHCs, each of whom had at least a year’s experience of supervising ASHAs. (Footnote to Fig. 1 for definitions of these roles.)

**Fig. 2:**
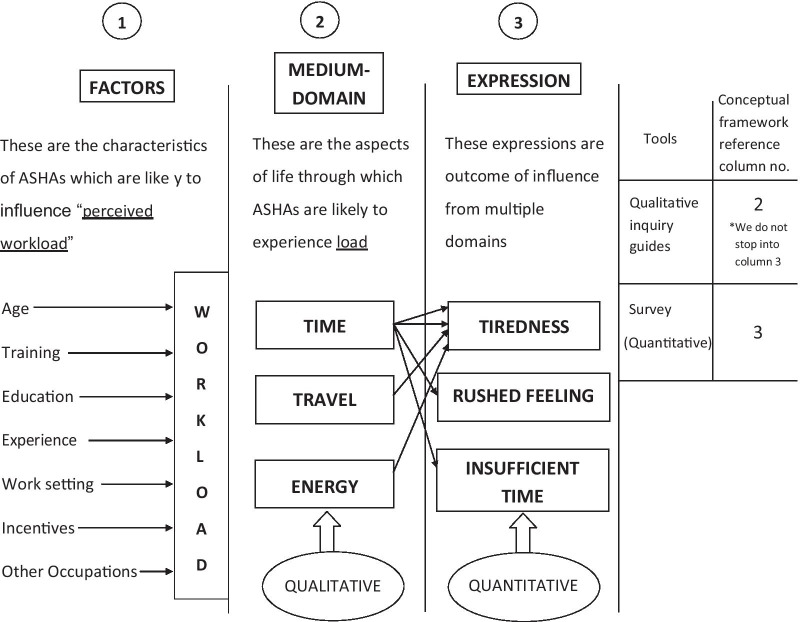
Initial conceptual framework

We developed a conceptual framework (Fig. 2) to inform the interview guide based on open-ended informal discussions with ASHAs and other colleagues. This helped us to understand the workload in terms of time investment, travel, energy and effect of the work on the ASHA’s family and self, and highlighted age, training, education, experience, work setting, incentives and other occupation as influencing factors. The volunteer status of ASHA was important in interpreting relative prioritisation of their work.

Topic guides were prepared in English (Additional file 2, Additional file 3) and translated into the local language (Marathi) by the Field Research Assistants during their training, reviewed by researchers, and piloted before finalisation. During data collection, the conversation focused on enquiry around typical daily activities. This led to probing on ASHA activities, household activities and other occupations, their perceptions of workload related to the different tasks, the challenges and motivations to continue the work. The qualitative interviews were conducted by graduate-level trained qualitative researchers, supported by a note-taker who had experience of working with the Health and Demographic Surveillance System (HDSS).

### Data handling and analysis

The quantitative data were analysed using Stata version15.0 and described as means and percentages. In-depth interviews were audio-recorded, transcribed verbatim into Marathi and translated into English by an independent qualitative researcher. Analysis, facilitated by MAXQDA version11.0 was undertaken by MG (anthropologist) supported by UC (health scientist) and PL (epidemiologist and qualitative researcher) who were also involved in data collection, cleaning and coding. Using an inductive approach, the transcripts were read repeatedly to identify frequently reported patterns related to objectives with similarities and differences. These were coded, categories developed, and emergent themes identified after discussion with wider team including an experienced anthropologist, medical and nursing experts. The data were triangulated using a ‘connecting data’ process [26] so that quantitative results were complemented by anecdotal evidence, reasons, examples and experiences from the qualitative data.

### Interpretation and stakeholder engagement

Throughout the data collection and analysis, the multidisciplinary team met regularly to discuss emerging themes and to ensure balanced interpretation mitigating reflexivity. Supported by the stakeholder engagement platform of RESPIRE (https://www.ed.ac.uk/usher/respire/about/supporting-platforms/platform-I-stakeholder-engagement-governance), we engaged with professional and lay stakeholders including the participating MO, ANM and BFF throughout the research process in order to broaden perspectives. A dissemination meeting was organised with all study participants and feedback encouraged.

## Results

### Participants’ characteristics

We recruited 67 ASHAs who completed the questionnaire; two-thirds were from rural area (*n* = 43) with more than half (*n* = 38) being in age group of 30–39 years. Almost all were married (*n* = 64) and 82% (*n* = 55) were engaged in family agricultural work in addition to ASHA work.

### Conceptual framework

**Fig. 3:**
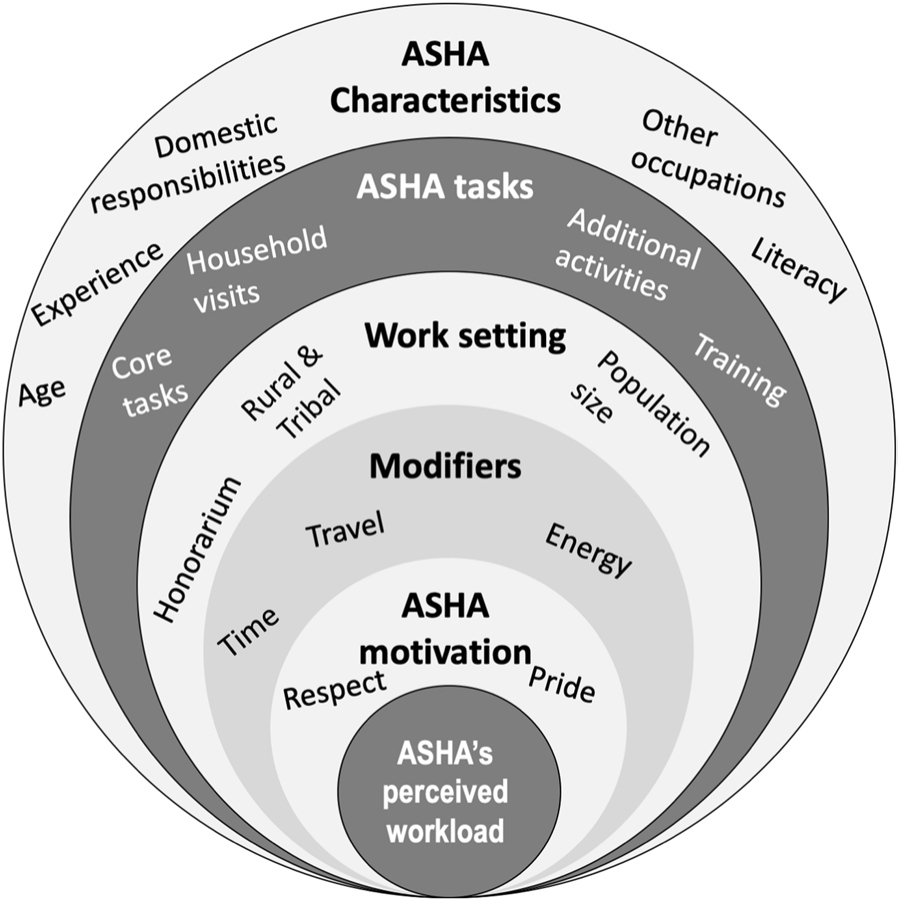
Schema for conceptual framework of ASHA workload perception

Figure 3 is the conceptual framework which illustrates our understanding of the ASHA’s perceived workload. The results are provided in Tables 1, 2, 3, 4, 5, with key findings presented in the text below. Perception of workload was influenced by multiple interacting factors such as characteristics, tasks, work settings and modified by time, travel, energy and motivation. Finally, we present the evolving nature of ASHA’s role.

**Table 1:** Demographic characteristics of study participants

Sr. no.	Variable	Total N* (%)	Rural *N* (%)	Tribal *N* (%)
		67	*N* = 43 (64)	*N* = 24 (36)
1	*Age*			
	< = 29	12 (17.9)	8 (18.6)	4 (16.6)
	30– < = 39	38 (56.8)	22 (51.2)	16 (66.7)
	40– < = 49	13 (19.4)	9 (20.9)	4 (16.6)
	50 and above	4 (5.9)	4 (9.3)	0
2	*Education*			
	Secondary school certificate (SSC)	48 (71.6)	25 (58.1)	23 (95.8)
	Higher secondary certificate (HSC)	13 (19.4)	12 (27.9)	1 (4.2)
	Graduate	5 (7.4)	5 (11.6)	0
	Postgraduate	1 (1.4)	1 (2.4)	0
3	Social category**			
	Open	37 (55.2)	35 (81.3)	2 (8.4)
	Other backward class (OBC)	5 (7.4)	5 (7.4)	0
	Nomadic tribes (NT)	1 (1.7)	1 (1.4)	0
	Scheduled caste (SC)	2 (2.9)	1 (1.4)	1 (4.1)
	Scheduled tribes (ST)	22 (32.8)	1 (1.4)	21 (87.5)
4	*Marital status*			
	Married	64 (95.6)	42 (97.6)	22 (91.6)
	Divorce	2 (2.9)	0	2 (8.4)
	Widow	1 (1.5)	1 (2.4)	0
5	*Other occupation*			
	Family agriculture work	55 (82.2)	32 (74.4)	23 (95.8)
	Home servant	2 (2.9)	1 (2.4)	1 (4.2)
	Labour work	1 (1.5)	1 (2.4)	0
	Personal business	6 (8.9)	6 (13.9)	0
	Seasonal	3 (4.5)	3 (6.9)	0
6	*Experience in years*			
	0–5 years	21 (31.4)	12 (27.9)	9 (37.5)
	6–10 years	37 (55.2)	31 (72.1)	6 (25)
	> 10 years	9 (13.4)	0	9 (37.5)

^*^One participant withdrew his participation after first questionnaire and hence *N* = 66 for tables hereafter

^**^Social categories are different categories in which the population is segregated depending on the caste system. As per policy of government of India the reserved categories; schedule caste (SC), schedule tribe (ST), nomadic tribe (NT), other backward class (OBC) gets some privileges such in employment and academic admissions. Population not falling in the reserve categories comes in open category [34]

**Table 2:** Interest to take up new tasks

Sr. no.	Variables	Total, *N* (%)	Rural, *N* (%)	Tribal, *N* (%)
1	*No. of activities last 6 months (including health-related activities, surveys and public health task)*			
	< = 5	32 (48.8)	12 (27.9)	10 (76.9)
	> 5 and < = 10	26 (39.1)	23 (53.1)	3 (23.1)
	> 10 and < = 15	8 (12.1)	8 (18)	0
2	*Possibility of new work*			
	Yes	62 (93.9)	40 (93)	22 (95.6)
	No	4 (6.1)	3 (7)	1 (4.4)

**Table 3:** Work settings

Sr. no.	Variable	Total *N* (%)	Rural *N* (%)	Tribal *N* (%)
1	*No. of villages served*			
	One	56 (84.8)	43 (100)	13 (56.5)
	More than one	10 (15.2)	0 (0)	10 (43.5)
2	*Population served*			
	1200–1800	12 (18.2)	12 (27.9)	0
	800–1200	29 (43.9)	27 (62.7)	2 (8.6)
	< 800	25 (37.9)	4 (9.4)	21 (91.4)
3	*Household visits/week*			
	< 20	18 (27.2)	10 (23.2)	8 (34.7)
	20–40	35 (53)	23 (53.6)	12 (52.1)
	> 40	13 (19.8)	10 (23.2)	3 (13.2)
4	*Visits to block facilitator per month*			
	1 to 4	51 (77.2)	33 (76.7)	18 (78.2)
	> 4	15 (22.8)	10 (23.3)	5 (21.8)
5	*Visits to auxiliary nurse midwife/month*			
	1 to 4	36 (54.6)	20 (46.5)	16 (69.5)
	> 4	30 (45.4)	23 (53.5)	7 (30.5)
6	*Incentives for ASHA work*			
	Less > (1500)	52 (78.2)	32 (74.2)	20 (86.9)
	Moderate (1500–3000)	11 (16.2)	8 (18.6)	3 (13.1)
	More < 3000	3 (4.6)	3 (6.2)	0

**Table 4:** Satisfied with ASHA work

Sr.	Variables	Total, *N* (%)	Rural, *N* (%)	Tribal, *N* (%)
1	*Satisfied with ASHA work*			
	Yes	62 (93.9)	39 (90.6)	23 (100)
	No	4 (6.1)	4 (9.4)	0
2	*Happy with ASHA work*			
	Yes	63 (95.5)	40 (93)	23 (100)
	No	3 (4.5)	3 (7)	0

**Table 5:** ASHAs’ perception of workload

Sr. no.	Variable	Total, *N* (%)	Rural, *N* (%)	Tribal, *N* (%)
1	*No. of hours per week on ASHA work*			
	< 12	19 (28.7)	13 (30.3)	6 (26)
	12 to 20	31 (46.9)	21 (48.8)	10 (43.4)
	> 20	16 (24.4)	9 (20.9)	7 (30. 6)
2	*Time spent on ASHA work each day*			
	Less (0–2 h) everyday	8 (12.2)	5 (11.6)	3 (13)
	Moderate (2–4 h) everyday	30 (45.4)	21 (48.8)	9 (39.5)
	More (above four hours) everyday	28 (42.4)	17 (39.6)	11(47.8)
3	*Travel time for ASHA work*			
	Less (0–1 h per day)	9 (13.1)	7 (16.3)	2 (8.7)
	Moderate (1–2 h per day)	25 (37.8)	20 (46.5)	5 (21.8)
	More (above 2 h per day)	32 (48.1	16 (37.2)	16 (69.5)
4	*Last week feel tired*			
	Yes	45 (68.2)	29 (67.5)	16 (69.5)
	No	21 (31.8)	14 (32.5)	7 (30.5)
5	*Reason for tiredness*			
	ASHA work	32 (71.1)	22 (75.8)	10 (62.5)
	Household activity	8 (17.7)	4 (13.6)	4 (25)
	Social activity	5 (11.2)	3 (10.6)	2 (12.5)
6	*Less time for family last week*			
	Yes	33 (50.0)	23 (53.4)	10 (43.4)
	No	33 (50.0)	20 (46.6)	13 (56.6)
7	*Reason for less time for family*			
	ASHA work	24 (72.7)	17 (73.9)	7 (70%)
	Household work	7 (21.3)	5 (21.7)	2 (20%)
	Other paid work	1 (3)	0	1 (10%)
	Social activity	1 (3)	1 (4.4)	0
8	*Not enough time for work last week*			
	Yes	32 (48. 5)	22 (51.1)	10 (43.4)
	No	34 (51.5)	21 (48.9)	13 (56.6)
9	*Reason for not enough time for work*			
	ASHA work	22 (68.8)	17 (77.2)	5 (50)
	Household work	6 (18.8)	2 (9)	4 (40)
	Other paid work	2 (6.2)	1 (4.8)	1 (10)
	Social activity	2 (6.2)	2 (9)	0
10	*Felt rushed last week*			
	Yes	43 (65.2)	29 (67.5)	14 (60.8)
	No	23 (34.8)	14 (32.5)	9 (39.2)
11	*Reason for felt rushed*			
	Asha work	36 (83.7)	26 (89.6)	10 (71.4)
	Household work	5 (11.7)	2 (6.8)	3 (21.4)
	Other paid work	1 (2.3)	1 (3.6)	0
	Social activity	1 (2.3)	0	1 (7.2)

## Influencing factors: characteristics of ASHA, their tasks and work settings

### Education, training and experience

There were significant differences between ASHAs from rural and tribal areas. (Table 1). Rural ASHAs had higher educational attainment (five were graduates) and most (81%) were from an open category compared to tribal ASHAs, most of whom were from scheduled tribes (foot note of Table 1 for definitions). A third of tribal ASHAs had more than 10 years of experience as against none from rural. The relatively poor literacy of some of the tribal ASHAs affected documentation and record keeping. This was highlighted by an ANM who noted that these ASHAs required additional assistance and time for tasks completion:

ASHAs with low education levels don’t always remember everything from the trainings given to them [ANM-1].

### Domestic and other occupations

Being married, many of ASHAs struggled to balance daily household chores and ASHA responsibilities. Most had additional jobs and seasonal work to supplement the family income which sometimes hampered routine ASHA tasks. Despite this, the BFF appreciated the work of tribal ASHAs who devoted considerable time to ASHA work.

### Willingness for additional activities

Although maternal and child health services were their primary work, during the preceding 6 months, 58/67 (88%) of ASHAs had carried out up to 10 additional activities (health surveys, epidemic survey, facilitating bank account opening for conditional cash transfer schemes, etc.). Despite time constrains and completion pressures, all of them were willing to take on new tasks (Table 2) with the hope of reasonable compensation and travel support.

We are willing to accept new workload but, of course, in return for handsome compensation, we have put forth the same demand for survey work also. Family members expect us to bring money for additional efforts we are putting [Rural ASHA-1].

The ANM supported ASHAs in taking up new activities especially if it didn’t involve much travel or didn’t disturb their existing schedule. One ANM stated:

Health authorities at the state level have instructed us not to pressurise ASHAs for additional work as their incentives are very low [ANM-2].

However, MO had explained them the voluntary nature of additional work.

Medical officers have told us in one of the training that that our work is voluntary and at any point of time we can refuse to take up any assigned tasks to us (Rural ASHA-2).

### Tasks of the ASHA

Table 3 shows that most ASHAs [56 (85%)] worked in their village of residence, though 10 (43%) tribal ASHAs had responsibility for an additional village. Workload, in terms of household visits (20–40 visits/week) and working hours (12–20 h) was similar in the two areas, though tribal ASHA had responsibility for smaller populations (< 800). ASHAs visited their BFF and ANM between one and four times a month.

### Work setting

Tribal ASHAs and the ANMs explained how the demography of remote populations affected workload and incentives. Urbanisation and migration meant young people had left the villages leading to loss of incentives from maternal and child health services. Although home visits were mandatory, when ASHAs working in remote areas were unable to do frequent home visits, then telephone follow-ups were acceptable. Some discrepancies were noted in workload assessment. A BFF estimated that ASHAs worked for 1 to 1.5 h daily (excluding Sundays). This comes to 6–9 h/week, in contrast to the survey findings that 46% of ASHA reported working 12–20 h/week (Table 5).This could be because of non-regular activities, e.g. ASHAs have to do home visits to facilitate scheme benefits to pregnant women such as “Pradhan Mantri Matru Vandana Yojana” (a national maternity benefit programme providing a cash incentive of INR 5000 (GBP £53.64) to pregnant women and lactating mothers in respect of the first living child of the family) [27].

### Honoraria and incentives

ASHAs received incentives for activities like family planning, antenatal and postnatal care, home based follow-up of new-born, immunisation, adolescent health, cancer surveys, etc. Almost all the ASHAs were happy and satisfied with these activities, despite most receiving monthly incentives less than INR 1500. (GBP £16.09) (Table 3) when compared with time required for allotted work. They were willing to do more for the benefit of the communities they served, but expected more incentives (*‘Mobadla’*: money earned against work). MOs and ANMs used incentives to motivate ASHA’s involvement in different types of activities. Some ASHAs suggested a monthly payment of INR 2000–5000 (GBP 21.40–53.60) could be reasonable.

I wish to do full time ASHA work only, but for that I need to get a fixed salary, I expect that government should really look into this matter. [Tribal ASHA-1].

The MOs, ANMs, and BFF supported the need to increase the honorarium to reward good work.

I advocate that the good work of ASHA should be awarded with increase in the honorarium [ANM-3].

## Modifying factors affecting perception of workload: time, travel and energy

Most ASHAs described time spent for ASHA activities as moderate (2–4 h/day), more so in tribal areas (Table 5). Two-thirds of ASHAs reported feeling tired and rushed in the previous week, mainly because of ASHA work. Almost three-quarters considered their ASHA role reduced the time they had to spend with their family and described how it encroached on time for other (paid) work.

The qualitative interviews explored a typical ASHA day. The results showed that they started early, completing household activities and then taking up ASHA duties. They had to leave home early when additional tasks (such as surveys, camps) were assigned to them periodically throughout the year.

This contrasts with perceptions of senior staff who considered that ASHAs spend 80% of their time for household work and give only 20% time to ASHA activities.

One of the MO has expressed this differently,

ASHA need to mingle with people in the neighbourhood and chat with them and simultaneously do the work which is challenging to meet with timings. [MO-1].

It was observed that during seasonal occupations (e.g. agriculture) ASHAs were unable to complete all activities.

The seasonal work hampers regular work of ASHA [ANM-4].

The residential status of ASHA was intended to limit travel time. However, villages located in mountainous areas were inaccessible with poor roads and lack of transport meant tribal ASHAs had to walk through the hills to reach remote settlements. Private vehicles had to be paid for from their own pockets, unless they were fortunate to get a free lift. Sometimes, antenatal visits to remote hamlets were skipped. ANMs were aware of these challenges. The BFF demanded bicycles for ASHAs who travelled daily more than six kilometres.

## Motivation of ASHA: pride and respect

### Evolution of motivation for undertaking ASHA work

Most ASHAs were content with their job and “proud” of their work. They believed their work was “social work” for a good cause, beneficial to the community. Their attitude towards work was good; they worked happily and were committed to the role. Villagers appreciated their hard work. This trust helped them to gain entry to homes. ASHAs were considered as family members with whom even sensitive information could be shared.

I feel proud (abhimaan) to work as ASHA worker. I feel I am doing social work and feel satisfied. I do not have any problem about payment, but I get an opportunity to do social work [Rural ASHA-3].

Personal circumstances did not stop performing ASHA work. In one situation,

I was in an emotional turmoil for a month after my sister’s death, but I continued with the duties even then. [Rural ASHA-4].

She further recognised that people acknowledged her efforts to bring positive change to health care in the hamlet and proudly stated:

Now women go to hospitals for deliveries. [Rural ASHA-4].

Though envisaged as ‘activists’ ASHAs had started considering themselves as ‘workers. This was apparent in the terminology used. In none of the interviews was the word ‘activist’ used. ASHA typically described themselves as ‘ASHA workers’:

Other women work in the farms only and are housewives, but I am an ASHA worker. I feel proud that I am engaged in government work also, I feel proud. [Tribal ASHA-2].

### Cordial team relationships

ASHAs had cordial relations with colleagues and senior staff. Although they followed instructions and fulfilled their expected duties, the ANMs and BFF were aware that ASHAs were voluntary workers and were in a position to refuse tasks, so they respected and supported them in personal matters.

We cannot act as bosses of ASHA, as they are voluntary workers. [BFF-1].

### Appreciation of efforts and good work by the community and team

ASHAs earned respect and recognition in their villages and were acknowledged for their clinical knowledge. They felt very proud about their work. One ASHA described how the training and experience had equipped her to manage the care of pregnant woman, referring the woman to the PHC and ensuring safe delivery. This gave great satisfaction and people’s blessings even though the remuneration was small. Tribal ASHAs were appreciated for the time devoted to work and efficient service provision and their social status haven’t shown any effect on their work or community interactions.

## Discussion

### Summary

ASHAs work up to 20 h a week in their village of residence, serving populations of about 800–1200 embracing an increasing range of activities despite a workload that contributed to feeling rushed and tired. Mitigating travel and time challenges, they have to prioritise between ASHA and other work. Despite less incentives, sense of benefiting the community and “pride of ASHA work” brought job satisfaction and happiness. Significantly, however, they described themselves as an “ASHA workers” and not as a “Health Activists”.

### Strengths and limitations

The study’s strength is the triangulation of data that enables qualitative exploration of the quantitative survey data about the breadth of work undertaken by contemporary ASHA. The multi stakeholder perspective (interviews with ASHAs, ANMs, BFFs and MOs) helped provide holistic understanding of the findings.

We were aware of reflexivity throughout the research process [28]. Data collection aimed to ensure construction of topics between researchers and participants. Meanings were negotiated and understood within the particular social context and validated in discussion with other researchers. The final interpretation was a consolidation of the perspectives of participants (at final feedback meeting), researchers, lay and professional stakeholders (involved with on-going project discussions) and the multidisciplinary research team.

Although our study was limited to two diverse areas in one state, we recruited all ASHAs in those areas and their demographic profile was similar to that of other studies. Our decision to purposively sample ASHA of more than 5 years of standing for the qualitative interview enabled us to gain perspectives from experienced ASHA who would have understood the evolution of the role, but meant that we do not have in-depth views of relatively new ASHA.

The data were collected during monthly meetings that enabled 100% participation, but this may have affected the answers as, despite reassurances of confidentiality, ASHAs may have been reticent to give their honest opinions as they knew the study was being promoted by their managers. ASHAs were given a private space to complete the questionnaires and asked to consult a study researcher (not a colleague) in case of any difficulty.

Interviews were conducted in Marathi, the language spoken and understood by ASHAs, though with a different dialect in rural and tribal areas. Interview guides were not back-translated due to lack of resources. English translations might have lost nuances of speech, though the researchers were fluent in both languages.

## Interpretation in the light of existing literature

### Characteristics of ASHA

As originally envisaged, ASHA must be the resident female volunteer of age 30–39 years, educated to at least 8th grade [4]. These selection criteria were fulfilled by rural ASHAs in our study and similar characteristics have been reported in the states of Madhya Pradesh and Uttar Pradesh [22, 23]. However, tribal ASHAs were younger and many were less educated; reflecting findings from the state of Gujrat [21] and Orissa who similarly showed some relaxation of these criteria [29]. This suggests our findings might have wider applicability within India and potentially in similar global contexts [30].

## Workload: time, travel and energy

Reflecting the remote topography and poor transport links, we observed significant differences between working arrangements in tribal settings compared to rural areas with respect to work area, number of villages and population served.

Village residence was intended to limit travel, but we found that in reality ASHAs have to travel regularly for home visits, camps, surveys, meetings, training, etc., and those from tribal areas had exhaustive travel as they served sparse populations in remote areas with poor transport. This echoes findings from a time-motion study from South India which found that travel encroached on the time tribal ASHAs spent performing duties [31]. We found that most ASHAs work in their own village serving populations of less than 1200. This is in contrast with populations as low as 454 persons/ASHA in Chhattisgarh to 1431 persons /ASHA in the State of Uttar Pradesh. Our survey reported working around 12 h/week and conducting 20 household visits /week, which is less than in Karnataka where, ASHAs worked for 3.8 h per day, covering a similar population but only undertaking 10 household visits per week [12]. Substantiating findings from other studies [15, 16, 20], our participants explained how they have to juggle their ASHA work with family commitments and other work, which contribute to feelings of tiredness and being rushed. The impact of lower educational status on efficiency in performing regular tasks is supported by a study in Rajasthan, India [24].

### Incentives

Many studies have reported the discontent over the small incentives and the demands for a simpler process of payment without any administrative delays [32]. Having a regular flow of funds is important to avoid demotivation [33]. In Punjab, incentives were found to be both empowering and conversely, a source of distress to ASHAs and family members. Incentives gave a sense of freedom, but the small, irregular and incomplete payments put pressure on families and was a major factor influencing prioritisation between ASHA activities and other paid jobs [20]. In Pakistan, Lady Health Workers have requested some stability of payment to sustain themselves [34]. Case studies from Iran, Ethiopia, India, Bangladesh and Nepal have reported that minimal incentives limit the focus of CHW work and improving the financial incentives results in the activities being prioritised [30]. In Orissa, a higher income and improved recognition contributed to feelings of self-efficacy [29]. Thus, in common with previous research [12, 17, 20, 29, 30, 34], although the ASHAs in our study were disappointed with incentives, they were generally happy and satisfied with the work and were motivated to continue.

## Evolution of the ASHA role

Perception of ASHA’s status and role was another potential line of discussion that emerged during interviews.

At its inception, it was envisaged that ASHAs would perform the roles of health facilitator, limited service provider (having drug kit to provide basic care and manage minor ailments) and health activists with the status of volunteers [4]. However, the different states have adapted the guidelines to suit their interpretation of the above roles which had affected the nature of support and training provided to ASHAs [12]. Though all the three roles are important and complementary for its’ effectiveness, ASHAs have been more successful in performing a link-worker role than community mobilisation or social determinants of health except for Chhattisgarh’s “*Mitanins”* (local word for CHW) who have performed as socio-political actors [13]. Rajasthan study showed that ASHA would prefer to choose on their ‘incentive-based’ role rather than ‘activist’ role [15]. Recently, in various states ASHAs’’ support has been taken for different work profiles/activities apart from ASHA’s traditional role in maternal and child care. Thus, ASHA’s changing role is highlighted in NCD [screening, prevention, and management], use of TeCHo model in Gujarat, [use of technology to improve performance], MAS—Mahila Arogya Samiti [improve immunisation coverage] and VHSNC—Village Health Sanitation Nutrition Committee [responding during natural disasters] in Odisha and the PFMS—Public Finance Management System [streamlining ASHA payments] in Assam [6]. Colloquial terminology, however, still describes ASHAs as ‘ASHA workers’ and not ‘Accredited Social Health Activists”. This reflects the move of many ASHAs to re-formulate their status as workers within the public health system, answerable to line managers such as ANM or MO. This change in perceived role has prompted some ASHAs to claim permanent jobs as employees of the health system [35]. Similar change in perception of role of CHW was seen in USA [36]. However, the impact of such a change on their pride and the respect that they gain in the community is not yet clear.

This evolution is apparent in other areas of India and other countries where CHWs have a pivotal role [37] In Pakistan, LHW wanted a position to meet their new aspirations [34]. Within culturally diverse regions of India, other studies have highlighted that the voluntary status of ASHA workers brought a sense of honour and motivation [12, 13]. In contrast, a study in a tribal area of Maharashtra reported that community did not always appreciate the voluntary status of ASHA, leading to some mistrust about their incentives which adversely affected the community response [38]. Our findings echo the changing and expanding role of CHWs (ASHAs) describing some of the issues related to their voluntary status.

## Implications of the study

Our findings have implications for advocacy and policy. Positive attitude of the ASHAs may be enhanced by providing them with predictable financial and non-financial incentives, ensuring frequent supportive supervision, and on-going training. At policy level, our findings align with, and shed some light on the evolution in the role of the ASHA. The ASHAs we spoke to are broadly satisfied with their current role (with some practical caveats), but in the future there may need to be a paradigm shift in the design of the ASHA programme reflecting the evolving role. Better entrench in the communities and coordination with health systems could help them evolve as an “activist”.

## Conclusion

Despite struggle between household commitments and ASHA activities, they are happy, satisfied and willing to take on new responsibilities. Future studies need to focus on developing strategies to recruit, train, incentivise, and retain ASHA. The voluntary status and activist role of ASHAs need to be understood and addressed if ASHAs are to be remain as a key in achieving universal health coverage in India.

## Supplementary Information

**Additional file 1:** Study questionnaire.

**Additional file 2:** Interview guide.

**Additional file 3:** Interview guide.

## Data Availability

The data that support study findings are available from the corresponding author on reasonable request.

## Abbreviations

ANM: Auxiliary nurse midwife
ASHA: Accredited Social Health Activists
BFF: Block facilitator female
CHW: Community health worker
LMIC: Low-middle income countries
MO: Medical officer
NCD: Non-communicable disease
PHC: Primary Health Centre
WHO: World Health Organization

## References

[1] Bhutta ZA, Lassi ZS, Pariyo G, Huicho L (2010). Global experience of community health workers for delivery of health-related millennium development goals: a systematic review, country case studies, and recommendations for integration into national health systems. Glob Health Workforce Alliance.

[2] Community health workers: what do we know about them? The state of the evidence on programmes, activities, costs and impact on health outcomes of using community health workers: Evidence and Information for Policy, Department of Human Resources for Health Geneva, WHO January 2007

[3] Javanparast S, Windle A, Freeman T, Baum F (2018). Community health worker programs to improve healthcare access and equity: are they only relevant to low-and middle-income countries?. Int J Health Policy Manag.

[4] Ministry of Health and Family Well-fare, Accredited Social Health Activist (ASHA) Operational Guidelines for ASHA under NRHM (2005). Accessed 24 Feb 2020.

[5] National Health Mission-MIS report as on Sept 2019.

[6] Update on ASHA program, July 2019, National Health Mission.

[7] Ministry of Health and Family Welfare-Update on ASHA program Jan 2017, National Health Mission. Accessed 1 Jan 2020.

[8] Evaluation of Accredited Social Health Activists (ASHA) Press Information Bureau Government of India Ministry of Health and Family Welfare. 27 Feb 2015. 12:

[9] Wagner AL, Porth JM, Bettampadi D, Boulton ML (2018). Have community health workers increased the delivery of maternal and child healthcare in India?. J Public Health.

[10] Evaluation of ASHA Program2010–11Executive_Summary. (n.d.). jhpn0033–0137. (n.d.).

[11] Perry H, Zulliger R, Scott K, Javadi D, Gergen J. Case studies of large-scale community health worker programs: examples from Bangladesh, Brazil, Ethiopia, India, Iran, Nepal, and Pakistan. Afghanistan: Community-Based Health Care to the Ministry of Public Health. 2013 Oct 28.

[12] Sundararaman T, Ved R, Gupta G, Samatha M. Determinants of functionality and effectiveness of community health workers: results from evaluation of ASHA program in eight Indian states. In: BMC proceedings 2012 Sep (Vol. 6, No. 5, p. O30). BioMed Central.

[13] Scott K, George AS, Ved RR (2019). Taking stock of 10 years of published research on ASHA programme: examining India’s national community health worker programme from a health systems perspective. Health Res Policy Syst.

[14] Survey International Institute of Population Sciences. National Family Health Survey (NFHS-4) Mumbai, India, https://dhsprogram.com/pubs/pdf/FR339/FR339.pdf.

[15] Sharma R, Webster P, Bhattacharyya S (2014). Factors affecting the performance of community health workers in India: a multi-stakeholder perspective. Glob Health Action.

[16] Saprii L, Richards E, Kokho P, Theobald S (2015). Community health workers in rural India: analysing the opportunities and challenges Accredited Social Health Activists (ASHAs) face in realising their multiple roles. Hum Resour Health.

[17] Paul D, Gopalakrishnan S, Singh P (2013). Functioning of accredited social health activists (ASHAs) in ICDS: an evaluation. Health Popul Perspect Issues.

[18] Kok MC, Broerse JE, Theobald S, Ormel H, Dieleman M, Taegtmeyer M (2017). Performance of community health workers: situating their intermediary position within complex adaptive health systems. Hum Resour Health.

[19] National programme for prevention and control of cancer, diabetes, cardiovascular diseases & stroke (NPCDCS) operational guidelines (revised: 2013–17), Directorate General of Health Services Ministry of Health & Family welfare Government of India 2013.

[20] Sarin E, Lunsford SS, Sooden A, Rai S, Livesley N (2016). The mixed nature of incentives for community health workers: lessons from a qualitative study in two districts in India. Front Public Health.

[21] Bansal SC, Nimbalkar SM, Shah NA, Shrivastav RS, Phatak AG (2016). Evaluation of knowledge and skills of home-based newborn care among accredited Social Health Activists (ASHA). Indian Pediatr.

[22] Waskel B, Dixit S, Singodia R, Pal DK, Toppo M, Tiwari SC, Saroshe S (2014). Evaluation of ASHA Programme in selected block of Raisen district of Madhya Pradesh under the National Rural Health Mission. J Evol Med Dent Sci.

[23] Shashank KJ, Angadi MM, Masali KA, Wajantri P, Bhat S, Jose AP (2013). A study to evaluate working profile of accredited social health activist (ASHA) and to assess their knowledge about infant health care. Int J Cur Res Rev.

[24] Karol GS, Pattanaik BK (2014). Community health workers and reproductive and child health care: an evaluative study on knowledge and motivation of ASHA (Accredited social health activist) Workers in Rajasthan, India. Int J Hum Social Sci.

[25] Srivastava SR, Srivastava PS (2012). Evaluation of trained Accredited Social Health Activist (ASHA) Workers regarding their knowledge, attitude and practice about child health. Rural Remote Health (online).

[26] Creswell JW, Klassen AC, Plano Clark VL, Smith KC. Best practices for mixed methods research in the health sciences. Bethesda (Maryland): National Institutes of Health. 2011; 2013:541–5.

[27] Ministry of Women and Child Development, Government of India, Pradhan Mantri Matru Vandana Yojana. www.wcd.nic.in. Accessed 12 Feb 2020.

[28] Finlay L (2002). “Outing” the researcher: the provenance, process, and practice of reflexivity. Qual Health Res.

[29] Gopalan SS, Mohanty S, Das A (2012). Assessing community health workers’ performance motivation: a mixed-methods approach on India's Accredited Social Health Activists (ASHA) program. BMJ Open..

[30] Singh D, Negin J, Otim M, Orach CG, Cumming R (2015). The effect of payment and incentives on motivation and focus of community health workers: five case studies from low-and middle-income countries. Hum Resour Health.

[31] Singh S, Upadhyaya S, Deshmukh P, Dongre A, Dwivedi N, Dey D, Kumar V (2018). Time motion study using mixed methods to assess service delivery by frontline health workers from South India: methods. Hum Resour Health.

[32] Bhatia K (2014). Stakeholders’ perspectives. Econ Polit Weekly.

[33] Regularization of NHM workers and ASHA in all over India. www.change.org. Accessed 26 Jun 2018.

[34] Khan MS, Mehboob N, Rahman-Shepherd A, Naureen F, Rashid A, Buzdar N, Ishaq M (2019). What can motivate Lady Health Workers in Pakistan to engage more actively in tuberculosis case-finding?. BMC Public Health.

[35] ASHA workers demand fixed pay; Hindu, 14th Jul 2020 https://www.thehindu.com/news/cities/Coimbatore/asha-workers-demand-fixed-pay/article32071639.ece?

[36] Malcarney M-B, Pittman P, Quigley L, Horton K, Seiler N (2017). The changing roles of community health workers. Health Serv Res..

[37] WHO guideline on health policy and system support to optimize community health worker programmes. Geneva: World Health Organization; 2018. ISBN 978-92-4-155036-9. 30431747

[38] Guha I, Raut AV, Maliye CH, Mehendale AM, Garg BS (2018). Qualitative Assessment of Accredited Social Health Activists (ASHA) Regarding their roles and responsibilities and factors influencing their performance in selected villages of Wardha. Int J Adv Med Health Res.

