# Supplementary material for: Interplaying role of healthcare activist and homemaker: a mixed-methods exploration of the workload of community health workers (Accredited Social Health Activists) in India
Source: Hum Resour Health. 2021 Jan 6;19:7. doi: 10.1186/s12960-020-00546-z (PMC7789492; doi:10.1186/s12960-020-00546-z)
Supplement: Supplementary file 1 — Additional file 1: Study questionnaire. [file 12960_2020_546_MOESM1_ESM.pdf]

ASHA workload study

Vadu Rural Health Program,
KEM Hospital Research Centre Pune and University of Edinburgh, United Kingdom

Start time: □□:□□	
Date: □□/□□/□□□□	
Interviewer Name	
Signature	
Participant’s Name	
Contact number	
Residence	
Type of Participant:	1. ASHA worker<br>~~2. Block facilitator~~<br>~~3. ANM~~<br>~~4. Medical officer~~<br>~~5. Community member~~
Date of Birth	□□/□□/□□□□
Age (in completed years)	
Education (in completed years)	
Name of PHC	
Type of PHC	1. Rural<br>2. Tribal
Name of Sub centre	

Work area		
Date of Joining	□□/□□/□□□□	
Duration of ASHA work experience	Days	□□
	Months	□□
	Years	□□
End time: □□:□□		

Sr. No.	Questions	Response categories	Code
1	Where is your work area?	1. Same Village<br>2. Another Village	
2	How many villages do you serve?	1. One<br>2. Two<br>3. Three<br>4. Four or more	
3	How much population do you serve?	5. One<br>6. Two<br>7. Three<br>8. Four or more	
4	How many houses do you visit per week?	1. Less than 800<br>2. 801-1200<br>3. 1201-1800<br>4. 1800 or more	
5	How many hours do you work per week?	1. Less than 20<br>2. 21-40<br>3. 40 or more	
6	How many times do you visit to BF per month?	1. 1 to 4 times<br>2. More than 4 times	
7	Number of visits to ANM per month?	1. 1 to 4 times<br>2. More than 4 times	
8	Your opinion about time requirement for ASHA work	1. Less<br>2. Moderate<br>3. More	
9	Your opinion about travel requirement for ASHA work	1. Less<br>2. Moderate<br>3. More	
10	Your opinion about monetary compensation requirement for ASHA work	1. Less<br>2. Moderate<br>3. More	
11	In last week, was there any day when you felt tired?	1. Yes<br>2. No → **(Go to question 13)**	

12	If Yes, what were the reasons?	1. Household work<br>2. Social activity<br>3. ASHA work<br>4. Other paid work<br>5. Any other activity<br>(Please mention)_______________________	
13	In last week, was there any day when you felt that you could not give sufficient time to your family members?	1. Yes<br>2. No → **(Go to question 15)**	
14	If Yes, what were the reasons?	1. Household work<br>2. Social activity<br>3. ASHA work<br>4. Other paid work<br>5. Any other activity<br>(Please mention)_______________________	
15	In last week, was there any day when you felt that you had insufficient time?	1. Yes<br>2. No → **(Go to question 17)**	
16	If Yes, what were the reasons?	1. Household work<br>2. Social activity<br>3. ASHA work<br>4. Other paid work<br>5. Any other activity<br>(Please mention)_______________________	
17	In last week, was there any day when you felt rushed?	1. Yes<br>2. No → **(Go to question 19)**	
18	If Yes, what were the reasons?	1. Household work<br>2. Social activity<br>3. ASHA work<br>4. Other paid work<br>5. Any other activity<br>(Please mention)_______________________	

19	Are you satisfied with ASHA work?	1. Yes<br>2. No	
20	Are you happy working as an ASHA worker?	1. Yes<br>2. No	
21	Which are the health activities and surveys you did in last 6 months?	1. __________<br>2. __________<br>3. __________<br>4. __________<br>5. __________<br>6. __________<br>7. __________<br>8. __________<br>9. __________<br>10. __________<br>11. __________<br>12. __________<br>13. __________<br>14. __________<br>15. __________	
22	Have you any experience of providing brief advice for smokeless tobacco cessation?	1. Yes<br>2. No	
23	If yes, were there any challenges in doing this? Please tell about those?	__________<br>__________<br>__________<br>__________	
24	What training would you need to deliver smokeless tobacco cessation advice?	__________<br>__________<br>__________<br>__________	
25	If new health activity is added to your ASHA work, will it be possible to manage within your current schedule?	1. Yes, possible<br>2. Yes, possible with some modification/alteration<br>3. Not possible	
**End time:** ☐☐:☐☐			
