# Supplementary material for: Interplaying role of healthcare activist and homemaker: a mixed-methods exploration of the workload of community health workers (Accredited Social Health Activists) in India
Source: Hum Resour Health. 2021 Jan 6;19:7. doi: 10.1186/s12960-020-00546-z (PMC7789492; doi:10.1186/s12960-020-00546-z)
Supplement: Supplementary file 2 — Additional file 2: Interview guide. [file 12960_2020_546_MOESM2_ESM.pdf]

ASHA workload study

PERCEPTIONS, EXPERIENCES AND ATTITUDES TOWARDS WORKLOAD
**SECTION 1: ABOUT GENERIC WORK**
➢ What is your concept of typical day?<br>➢ How do you start your day?<br>➢ What are other activities you do apart from household work?<br>➢ How much time is spent for this work?<br>➢ Do you do any other work apart from the work you mentioned earlier?
**SECTION 2**: ABOUT ASHA WORK
➢ What is your concept of typical day as an ASHA worker?<br>➢ What are the health activities you do as an ASHA worker?<br>➢ Which are the health activities you perform **daily?**<br>➢ Which are the health activities you perform **weekly?**<br>➢ Which are the health activities you perform **monthly?**<br>➢ Generally how many hours **daily** are spent in the **work area**?<br>➢ Generally how many **days in a month** are spent at **work area (or field)**?<br>➢ What is your opinion about **time** spent in the work area for actual health activities assigned to you?<br>➢ What is your opinion about **travel** for this work?<br>➢ What is your opinion about **other activities** involved in this work? e.g. report preparation
**SECTION 3:** Perceived burden of the work
➢ What is your opinion about current work assigned to you?<br>➢ Do you face any difficulties while doing ASHA work?<br>➢ Do you feel that you have sufficient **time** to manage all the activities?<br>➢ What is your opinion about **interpersonal relationship**(with community, ANMs, medical officers etc.)?<br>➢ What is your opinion about **monetary compensation** about ASHA work?<br>➢ In comparison with other ASHA workers are there any differences in the **setting at work area** which have influence on the ASHA work?<br>➢ Have you ever felt that you could not complete any task? If so, what were the reasons?<br>➢ Have you ever felt that you are overburdened with the work? If so, what were the reasons?
**SECTION 4:** How much more workload can be given?
➢ What is your opinion if new health activity is added to your current work? Do you think that new activity will add burden to your current work?<br>➢ Will you be able to manage new health activity (in terms of time and travel) along with your current work?<br>➢ Can you tell us some of the factors which would help you in performing new activity?<br>➢ How will you manage ASHA work along with your household work?<br>➢ How do you see yourself in comparison with other individuals in your surroundings in terms of **work load?**<br>➢ How do you see yourself in comparison with other individuals in your surroundings in terms of **time?**

➢ How do you see yourself in comparison with other individuals in your surroundings in terms of **travel?**<br>➢ How do you see yourself in comparison with other individuals in your surroundings in terms of **monetary compensation/financial benefit?**
SECTION 5: About smokeless tobacco cessation
➢ Have you any experience of providing brief advice for smokeless tobacco cessation?<br>➢ If yes, were there any challenges in doing this? Please tell about those?<br>➢ What training would you need to deliver smokeless tobacco cessation advice?
