# Supplementary material for: Interplaying role of healthcare activist and homemaker: a mixed-methods exploration of the workload of community health workers (Accredited Social Health Activists) in India
Source: Hum Resour Health. 2021 Jan 6;19:7. doi: 10.1186/s12960-020-00546-z (PMC7789492; doi:10.1186/s12960-020-00546-z)
Supplement: Supplementary file 3 — Additional file 3: Interview guide. [file 12960_2020_546_MOESM3_ESM.pdf]

ASHA workload study

For Medical officers, ANMs and ASHA supervisors
**SECTION 1:**<br>Perceptions, experiences and attitudes towards workload
GENERAL<br>➢ What are the health activities are carried out by ASHAs?<br>➢ Which are the health activities that need to be done on **daily basis?**<br>➢ Which are the health activities that need to be done on **weekly basis?**<br>➢ Which are the health activities that need to be done on **monthly basis?**<br>➢ As per your opinion on average how many **hours per day** are spent for this work?
TIME<br>➢ Generally how many **days in a month** are spent at **work area (or field)** by ASHAs?<br>➢ Generally how many hours **daily** are spent in the **work area** by ASHAs?<br>➢ Generally how many hours **weekly** are spent in the **work area** by ASHAs?<br>➢ Generally how many hours **monthly** are spent in the **work area** by ASHAs?<br>➢ Generally how many **days in a month** are spent at **sub centre** by ASHAs?<br>➢ Generally how many **days in a month** are spent at **PHC** by ASHAs?
TRAVEL<br>➢ How frequently they need to travel to the **work area**?<br>➢ How frequently they need to travel to the **sub centre**?<br>➢ How frequently they need to travel to the **PHC**?<br>➢ Generally **how many days in a month** they have to travel as a part of ASHA work?
OTHER<br>➢ How frequently ASHAs have to attend meetings?<br>➢ How frequently ASHAs have to attend trainings?<br>➢ How frequently ASHAs have the documentation work (e.g. report preparation)?<br>➢ What is your opinion about ASHAs visits for follow up of with the beneficiaries/ Revisit to the beneficiaries?
MONETARY COMPENSATION<br>➢ What is your opinion about compensation for ASHA work?<br>➢ Is there relation between an attention paid towards health activity and provided compensation?<br>➢ Did you ever feel that there is controversy in the work and provided compensation?
**SECTION 2**: ABOUT CURRENT WORKLOAD
➢ What is your opinion about **time** spent in the work area for actual health activities by ASHAs?<br>➢ What is your opinion about **travel** for this work?<br>➢ What is your opinion about **other activities** involved in this work?(e.g. report preparation)?

ASHA workload study

SECTION 3: Perceived burden of the work

- What is your opinion about current workload of ASHAs?
- Do you feel that they have sufficient time to manage all the activities?
- Can you tell some of the reasons where ASHAs could not complete any task?
- What is your opinion about interpersonal relationship (with community, ANMs, medical officers etc.) and burden of the work?
- Do you see any difference in the work due to differences in area situation (or work setting)?

SECTION 4: How much more workload can be given?

- What is your opinion if new health activity is added to their current work?
- Will they be able to manage new health activity (in terms of time and travel) along with your current work?
- Can you tell us some of the factors which would help them in performing new activity?
